# Regulation of TIA-1 Condensates: Zn^2+^ and RGG Motifs Promote Nucleic Acid Driven LLPS and Inhibit Irreversible Aggregation

**DOI:** 10.3389/fmolb.2022.960806

**Published:** 2022-07-14

**Authors:** Danella L. West, Fionna E. Loughlin, Francisco Rivero-Rodríguez, Naveen Vankadari, Alejandro Velázquez-Cruz, Laura Corrales-Guerrero, Irene Díaz-Moreno, Jacqueline A. Wilce

**Affiliations:** ^1^ Monash Biomedicine Discovery Institute and Department of Biochemistry and Molecular Biology, Monash University, Clayton, VIC, Australia; ^2^ Institute for Chemical Research, University of Seville—CSIC, Seville, Spain

**Keywords:** TIA1, RNA binding protein, liquid-liquid phase separation, prion-like domain, RRM, amyloid fibril, zinc, RGG motif

## Abstract

Stress granules are non-membrane bound RNA-protein granules essential for survival during acute cellular stress. TIA-1 is a key protein in the formation of stress granules that undergoes liquid-liquid phase separation by association with specific RNAs and protein-protein interactions. However, the fundamental properties of the TIA-1 protein that enable phase-separation also render TIA-1 susceptible to the formation of irreversible fibrillar aggregates. Despite this, within physiological stress granules, TIA-1 is not present as fibrils, pointing to additional factors within the cell that prevent TIA-1 aggregation. Here we show that heterotypic interactions with stress granule co-factors Zn^2+^ and RGG-rich regions from FUS each act together with nucleic acid to induce the liquid-liquid phase separation of TIA-1. In contrast, these co-factors do not enhance nucleic acid induced fibril formation of TIA-1, but rather robustly inhibit the process. NMR titration experiments revealed specific interactions between Zn^2+^ and H94 and H96 in RRM2 of TIA-1. Strikingly, this interaction promotes multimerization of TIA-1 independently of the prion-like domain. Thus, through different molecular mechanisms, these stress granule co-factors promote TIA-1 liquid-liquid phase separation and suppress fibrillar aggregates, potentially contributing to the dynamic nature of stress granules and the cellular protection that they provide.

## Introduction

Stress granules protect the cell during times of acute stress by sequestering mRNAs in dynamic RNA-protein compartments where they are protected from degradation (Riggs et al., 2020). Formation of stress granules occurs through a summation of multivalent protein and RNA interactions resulting in de-mixing from the cytosol through the physical process of liquid-liquid phase separation (LLPS) (Hofmann et al., 2021). The granules form with a dense core and a liquid-like shell, and remain in a dynamic assembled state for several hours until disassembly occurs (Wheeler et al., 2016). Aberrant stress granule dynamics is associated with neurodegenerative diseases, including amyotrophic lateral sclerosis (ALS), fronto-temporal lobar degeneration (FTLD) as well as tauopathies (Alberti and Dormann, 2019; Wolozin and Ivanov, 2019; Alberti and Hyman, 2021). Specifically, delayed disassembly and reduced dynamics within stress granules is widely hypothesized to facilitate the aggregation of stress granule-associated proteins, resulting in pathologic inclusions that are a hallmark of these neurodegenerative diseases (Zhang et al., 2019).

TIA-1 is a characteristic stress granule protein that binds mRNA and contributes to stress granule formation *via* its C-terminal Prion-like domain (PrLD) (Gilks et al., 2004). The N-terminus of TIA-1 comprises three RRM domains, of which RRM2 and 3 (“RRM2,3”) cooperatively bind target RNA with high affinity (Dember et al., 1996; Cruz-Gallardo et al., 2014; Waris et al., 2017). *In vitro* studies of TIA-1 identified its intrinsic ability to undergo LLPS either alone (Mackenzie et al., 2017) or as enhanced by multi-site target nucleic acid (Loughlin et al., 2021). These same studies also showed that, over time, TIA-1 progresses to form irreversible *ß*-sheet rich aggregates and that this is also enhanced in the presence of target nucleic acid. Thus, the self-associating properties that underlie TIA-1 function also predispose it to irreversible aggregation, potentially underlying disease. In fact, mutations in TIA-1 associated with Welander distal myopathy (WDM) and ALS confer a greater propensity for aggregation and delayed stress granule disassembly, consistent with this mechanism for disease (Mackenzie et al., 2017). However, extensive amyloid aggregates of TIA-1 are not a distinctive feature of stress granules in healthy cells, suggesting that interactions preventing aggregation of TIA-1 protein exist within the cell. Herein we investigate the effect of stress granule co-factors on LLPS and fibril formation of TIA-1 *in vitro*.

Stress granules are made up of hundreds of proteins and co-factors, a subset of which specifically contribute to the assembly and maintenance of the liquid state (Jain et al., 2016; Tauber et al., 2020). Zn^2+^ has been reported to be a second messenger, enhancing the formation of TIA-1 positive stress granules (Rayman et al., 2018). The release of intracellular Zn^2+^ was observed in cells and tissues exposed to arsenite treatment, consistent with Zn^2+^ playing a role in stress granule formation in response to oxidative stress. When Zn^2+^ was made unavailable *in vivo* by chelation, the formation of TIA-1 positive stress granules was reduced (Rayman et al., 2018; Carrascoso et al., 2019). Studies of purified TIA-1 protein showed Zn^2+^ dependent multimerization using a Förster resonance energy transfer (FRET) coinciding with formation of LLPS without extensive *ß*-sheet formation (Rayman et al., 2018). This study thus suggested that a direct interaction between Zn^2+^ and TIA-1, and subsequent change in multimerization leading to LLPS, could influence stress granule formation. This raises questions of how Zn^2+^ differentially affects TIA-1 LLPS *vs* fibrillar aggregation, and the molecular basis of the Zn^2+^-TIA-1 interaction. In the current study, therefore, we analysed the effect of Zn^2+^ on LLPS and fibrillar aggregation of TIA-1, the nucleic acid-induced TIA-1 condensates and also investigated the molecular basis of the Zn^2+^:TIA-1 interaction.

Stress granules are particularly enriched with RNA-binding proteins with low complexity regions, including PrLDs and also another type of intrinsically disordered region (IDR) rich in Arg-Gly-Gly repeats (RGG) (Chong et al., 2018; Youn et al., 2019). RGG-containing proteins include the core stress granule protein G3BP1/2, in which the RGG region mediates an RNA induced switch to instigate condensation (Guillen-Boixet et al., 2020; Sanders et al., 2020; Yang et al., 2020), and the FUS protein, that is recruited to stress granules by its RGG-zinc finger domain in disease (Bentmann et al., 2012). It has also been shown that the Arg-rich repeat peptides derived from pathogenic C9orf72 expansions associate with stress granules, affecting their dynamics (Boeynaems et al., 2017). While well known for their ability to interact directly with RNA, RGG-rich IDRs can also interact with PrLDs, enhancing LLPS (Kaur et al., 2021). Multivalent transient interactions between the PrLD and RGG IDRs of FUS enhance phase separation (Qamar et al., 2018; Wang et al., 2018). Furthermore, ensuing condensates can co-recruit other RNA-binding proteins, including TIA-1, potentially also *via* intermolecular heterotypic RGG:PrLD interactions (Qamar et al., 2018; Wang et al., 2018). Thus, interactions between PrLD and RGG-rich IDRs are likely of fundamental importance in stress granule biology and disease. We have hence examined the interactions of the native RGG-rich RNA-binding domain of FUS (FUS-RBD) with TIA-1 as a model of the effect of heterotypic interactions in protein-RNA condensates.

The current study investigates the way in which stress granule proteins and co-factors influence LLPS and fibrillar aggregation of TIA-1. We report the impacts of Zn^2+^ and FUS-RBD on TIA-1 in the absence and presence of nucleic acid. Our investigation utilises *in vitro* assays of TIA-1 LLPS and fibrillar aggregate formation as previously used to probe the effect of nucleic acid (Loughlin et al., 2021) as well as NMR spectroscopy and site directed mutagenesis to further identify Zn^2+^ binding sites. These experiments revealed that the metal ion Zn^2+^ not only enhances LLPS of TIA-1 alone, but also further enhances nucleic acid-induced TIA-1 LLPS. NMR spectroscopy and mutational studies defined a zinc binding site on RRM2 leading to the identification of H94 and H96 as Zn^2+^ ligands. Surprisingly, Zn^2+^ was found to induce multimerization of the isolated TIA-1 RRM2,3 domains even in the absence of the PrLD, potentially reflecting an additional mode of TIA-1 self-interaction via Zn^2+^ ligation. Zn^2+^-enhanced LLPS of full length TIA-1, however, did not result in fibrillar aggregate formation of TIA-1 and Zn^2+^ strikingly inhibited nucleic acid-induced fibrillar aggregation of TIA-1 even at sub-stoichiometric levels. FUS-RBD also further enhanced nucleic acid-induced LLPS of TIA-1, but effectively inhibited fibrillar aggregation, presumably through a direct competition between PrLD:PrLD and PrLD:RGG interactions. Together, this study showcases two types of heterotypic interaction of TIA-1 that promote LLPS, but suppress the aggregation capacity of TIA-1 *in vitro*, potentially contributing to the maintenance of the reversible and dynamic state of TIA-1 within stress granules.

## Materials and Methods

### Cloning of TIA-1 RRM2,3 His Mutants

To construct the plasmid for expression of TIA-1 RRM2,3 (93-274), pGEX-4T-2 (GE Healthcare) was modified to bear a 6xHis tag and a thrombin cleavage site. TIA-1 RRM2,3 was inserted directly after the thrombin site using oligonucleotides TIA-1 RRM2,3-For (GCG​TGG​ATC​CCC​AGG​AAT​TCC​CAA​TCA​TTT​CCA​TGT​CTT​TGT​TGG​TG) and TIA-1 RRM2,3-rev (CAG​TCA​CGA​TGC​GGC​CGC​TTA​TTT​GCC​CCA​ATA​GCA​TTT​CAC​AAC). To construct the plasmid TIA-1 RRM2,3 bearing mutations H94A, H96 A and H94A H96A, site-directed mutagenesis by inverse PCR (Liu and Naismith, 2008) was performed using the oligonucleotide pairs:

TIA-1 RRM2,3-H94A-For: CCA​ATg​ctT​TCC​ATG​TCT​TTG​TTG​GTG​ATC​TCA​GCC​CAG

TIA-1 RRM2,3-H94A-Rev: ATG​GAA​agc​ATT​GGG​AAT​TCC​TGG​GGA​TCC​ACG

TIA-1 RRM2,3-H96A-For: TTT​Cgc​tGT​CTT​TGT​TGG​TGA​TCT​CAG​CCC​AG

TIA-1 RRM2,3-H96A-Rev: AAA​GAC​agc​GAA​ATG​ATT​GGG​AAT​TCC​TGG​GGA​TCC​ACG

TIA-1 RRM2,3-H94A H96A-For: CCA​ATg​ctT​TCg​cTG​TCT​TTG​TTG​GTG​ATC​TCA​GCC​CAG

TIA-1 RRM2,3-H94A H96A-Rev: AAA​GAC​agc​GAA​agc​ATT​GGG​AAT​TCC​TGG​GGA​TCC​ACG​CGG

### Full-Length TIA-1 Protein Preparation

TIA-1 protein short isoform (P31483-2) was expressed from plasmid pETM11 as a 6xHis-tagged protein in BL21 pLysS *E. coli* cells and purified as previously reported (Loughlin et al., 2021). In brief, this involved initial purification using Co-TALON metal affinity resin (Takara), followed by cleavage of the 6xHis-tag by using TEV protease and its removal using Ni-NTA resin. The final purification was by size exclusion chromatography in a buffer established to maintain TIA-1 solubility (20 mM sodium phosphate, 60 mM KCl, 0.5 M arginine-HCl, 1 mM MgCl_2_, 2 mM DTT, 0.5 mM EDTA, pH 7.0). Aliquots were concentrated to a maximum of 100 μM, filtered and stored at −80°C in size exclusion buffer. TIA-1 protein used in Zn^2+^ experiments was prepared in the same manner, with the exception that EDTA was not included in the final size exclusion step. The A_280/260_ ratio for each preparation was <0.65 AU and protein was quantified using a theoretical molar extinction coefficient of *80,330 M* ^*−1*^ *cm* ^*−1*^_*.*_

### TIA-1 RRM1 and RRM2,3 Preparation

GST-TIA-1 RRM1 (amino acids 1-81; in plasmid pGEX-4T-2) and 6xHis-TIA-1 RRM2,3 (amino acids 93-27; in plasmid pETtrx-1a) variants (Waris et al., 2017) were produced in *E. coli* BL21 (DE3) cells grown at 37°C to an OD_600_ of 0.6–0.8. LB medium was used if proteins were intended for turbidity measurements, whereas ^15^N-labelled TIA-1 constructs for NMR titrations were produced in M9 minimal medium with ^15^NH_4_Cl as a nitrogen source. Expression was induced by the addition of 1 mM IPTG and cultures were incubated at 30°C for 16–18 h. Cell pellets were resuspended in lysis buffer (20 mM potassium phosphate, 500 mM KCl, pH 7.4) with 20 μg/ml DNase I, 1 mM phenylmethanesulfonyl fluoride (PMSF) and 100 μg/ml lysozyme. Cells were disrupted by sonication (cycles of 30 s at 40% of amplitude, 60 s of rest, 6 min total time, on ice), and cell debris was removed by centrifugation at 28,000 × g (4°C for 45 min). The supernatant from GST-TIA-1 RRM1 was loaded onto a 5 ml Profinity GST column (BioRad) and eluted in a single-step with lysis buffer containing 20 mM GSH in a NGC Chromatography System Quest 10 (BioRad). The supernatant from 6xHis-TIA-1 RRM2,3 WT or its mutant variants was loaded onto a Ni-NTA matrix (ThermoFisher), previously equilibrated with lysis buffer containing 5 mM imidazole, and incubated for 1 h at 4°C. Recombinant proteins were eluted using a non-continuous imidazole gradient and purity was further checked by SDS-PAGE. The GST- and 6xHis-tag were removed by overnight incubation (4°C) of the protein with 2.5 U/mg of thrombin protease (Cytiva). Cleaved TIA-1 constructs were then isolated with Ni-NTA resin (for RRM2,3) or a two-step chromatography with ENrich SEC 650 (BioRad) and Profinity GST columns (for RRM1). Purified proteins were dialyzed twice against 5 L of either HEPES buffer (20 mM HEPES, 50 mM NaCl, pH 6.9) or citrate buffer (20 mM citrate, 50 mM NaCl, pH 5.5) at 4°C overnight. For complete disulphide bond reduction, buffered TCEP was added to dialyzed proteins up to a concentration of 1 or 5 mM for turbidity assays and NMR titrations, respectively. Then, samples were concentrated using Amicon Ultra-15 centrifugal filters (Merck-Millipore). Proteins were quantified by spectrophotometry at 280 nm using extinction coefficients of 8,480 M^−1^cm^−1^ for TIA-1 RRM1 and 30,940 M^−1^cm^−1^ for TIA-1 RRM2,3.

### FUS-RBD Protein Preparation

Two constructs of FUS were expressed and purified as per Loughlin et al. (Loughlin et al., 2019). FUS-RBD (amino acids 242-526) comprising FUS RGG1-RRM-RGG2-ZnF-RGG3 domains was produced from pET24b consisting of a N-terminal GB1 solubility tag followed by a 6xHis-tag. FUS-RBD-ΔRGG1/3 (amino acids 269-454) was produced from pET28a consisting of a N-terminal 6xHis tag. Each construct was expressed in *E coli* BL21 Rosetta2 cells grown at 37°C, followed by protein expression induction with 0.5 mM IPTG and overnight expression at a reduced temperature of 25°C. Cell pellets were resuspended in lysis buffer (50 mM Tris-HCl, 1 M NaCl, 0.5% Triton-X, 5 mM imidazole, 0.5 mM *ß*-mercaptoethanol, pH 8.0) and lysed by sonication. Lysate was clarified at 48,000 × g for 30 min, loaded onto Ni-NTA beads, washed with 20 and 40 mM imidazole in wash buffer (50 mM Tris-HCl, 1 M NaCl, 0.5 mM *ß*-mercaptoethanol, pH 8.0) and eluted with 200 mM imidazole in wash buffer. For FUS-RBD short, the 6xHis-tag was cleaved with TEV protease in 50 mM Tris-HCl, 1 M NaCl, 10 mM imidazole, 0.5 mM *ß*-mercaptoethanol, pH 8.0, and removed with Ni-NTA beads. Proteins were dialysed into storage buffer (50 mM Tris-HCl, 1 M NaCl, 20 mM imidazole, 0.5 mM *ß*-mercaptoethanol, pH 8), concentrated to 0.4 mM and stored at −80°C. Proteins were quantified using extinction coefficients of 36,883 M^−1^cm^−1^ for GB1-6xHis-FUS-RBD fusion and 20,970 M^−1^cm^−1^ for FUS-RBD-ΔRGG1/3.

### Oligonucleotides

Synthetic single stranded DNA oligonucleotides containing three or five TC-rich TIA-1 binding sites were synthesized and HPLC purified commercially (IDT, Australia) and quantified using molar extinction coefficients supplied by IDT.

TC3 (34 nt): TTT​TTA​CTC​CAA​TTT​TTA​CTC​CAA​TTT​TTA​CTC​C

TC5 (58 nt): TTT​TTA​CTC​CAA​TTT​TTA​CTC​CAA​TTT​TTA​CTC​CAA​TTT​TTA​CTC​CAA​TTT​TTA​CTC​C

### Turbidity Measurements

Turbidity measurements of full length TIA-1 were used to measure the relative amounts of LLPS under set conditions. Measurements were taken within 20 min of instigating LLPS, a timeframe in which we do not observe aggregation either by microscopy or ThT fluorescence. TIA-1 protein in size exclusion buffer was diluted either alone, or in the presence of nucleic acids in aggregation buffer. In general, TIA-1 protein was diluted to 2.5 μM in the presence of 0.5 μM oligonucleotides unless otherwise stated. Final aggregation buffer was 20 mM HEPES, 50 mM NaCl, pH 7.2, including residual 15 mM arginine-HCl. As controls, nucleic acid or ZnCl_2_ alone were diluted in aggregation buffer. Triplicate samples of 150 µL were set-up at room temperature and incubated for 10 min at 25°C in 96-well clear bottom non-binding black plates (Greiner), then analysed at 385 nm using a CLARIOstar plate reader (BMG Labtech). Assays included 3 replicates with error bars representing S.D.

Turbidity measurements of TIA-1 RRM2,3 WT, H94A, H96A and H94A H96A were performed at a final concentration of 20 μM in either HEPES buffer (20 mM HEPES, 50 mM NaCl, pH 6.9) or citrate buffer (20 mM citrate, 50 mM NaCl, pH 5.5). Each construct was measured at 25°C every min for 30 min after the addition of ZnCl_2_ or TPEN at the indicated ratios. Independent samples of 100 µL were prepared at room temperature and measured at 385 nm in a 96-well plate with a Varioskan LUX microwell plate reader (ThermoFisher). Graphs represent the average of the last 10 values of turbidity measurements, after signal stabilization. Assays included 3 replicates with error bars representing S.D. Boxplots were generated using R version 4.0.5 (http://www.r-project.org).

### DIC Microscopy

Samples of full length TIA-1 with or without ZnCl_2_ or oligonucleotides were prepared fresh as per samples used in turbidity measurements and imaged within 20 min of mixing unless otherwise stated. A 10 μL-aliquot of each sample was spotted onto a glass microscope slide with double sided tape and covered with 0.17 mm HP glass coverslip (Zeiss). Solutions were imaged at room temperature with differential interference contrast (DIC) on an Inverted Olympus IX81 x 2UCB microscope (Olympus, Tokyo, Japan) with a 60 × objective. Images were processed using FIJI (Schindelin et al., 2012).

### Thioflavin T Assay

Thioflavin-T (ThT) fluorescence assays were used to monitor ThT positive aggregate formation of full length TIA-1 alone and in the presence of nucleic acids. Samples were prepared as per turbidity assays in aggregation buffer. After turbidity measurements, a 1 mM ThT stock solution was diluted to a final concentration of 5 μM and equilibrated to 30 ^o^C for several minutes prior to measurements commencing. Samples were then agitated through orbital shaking at 500 r.p.m. and 30°C over 12–16 h, and ThT fluorescence was monitored with an excitation wavelength of 425 nm and an emission wavelength of 485 nm, using a CLARIOstar plate reader (BMG Labtech). Measurements were baseline corrected using ThT fluorescence of aggregation buffer/ThT alone. Assays included 3 replicates with error bars representing S.D.

### Transmission Electron Microscopy

To analye the morphology of the aggregates present at the endpoint of the ThT assay, samples were imaged by Transmission Electron Microscopy (TEM). A 5 µL of endpoint sample was applied to the surface of glow-discharged continues carbon grids and stained with 2% uranyl acetate solution. The excess stain was removed using filter paper and grids were air-dried. All samples were imaged on an FEI Tecnai F2 F20 TWIN electron microscope with a Gatan 4k x 4k CCD, under a working voltage of 200 kV at the Ramaciotti Centre for Cryo-Electron Microscopy of Monash University.

### NMR Measurements

Nuclear Magnetic Resonance (NMR) titrations involving TIA-1 constructs were recorded and monitored at 25°C by 1D ^1^H and 2D [^1^H-^15^N] Heteronuclear Single Quantum Correlation (HSQC) spectra in a Bruker Avance-III 700 and 500 MHz equipped with a 5 mm TCI cryoprobe. Samples were dialyzed in 20 mM citrate, 50 mM NaCl, 5 mM TCEP, pH 5.5, or 20 mM HEPES, 50 mM NaCl, 5 mM TCEP, pH 6.9, for NMR titrations. 5% D_2_O was added to all samples to adjust the lock signal of the NMR spectrometer. Samples were prepared at 200 or 300 μM for TIA-1 RRM1 and TIA-1 RRM2,3, respectively, in a final volume of 350 μL. Samples were loaded into Shigemi tubes (Shigemi Inc.). Data were acquired and processed using TopSpin 3.5pL7 software (Bruker). Linewidth broadening and chemical-shift perturbation analysis were performed using NMRFAM-SPARKY software distribution (National Magnetic Resonance Facility, Madison). The NMR assignment of TIA-1 RRM1 and TIA-1 RRM2,3 was already available (Biological Magnetic Resonance Bank [BMRB] accession numbers 34144 and 19,735, respectively) (Wang et al., 2014; Sonntag et al., 2017). Chemical-shift perturbations (Δδ_AVG_) were calculated as previously described (Rivero-Rodríguez et al., 2021).

### CD Spectropolarimetry

Circular Dichroism (CD) spectra were recorded in the far-UV range (195–250 nm) at 20°C on a Jasco J-815 CD spectropolarimeter equipped with a Peltier temperature control system. 10 µM of each TIA-1 RRM2,3 construct in phosphate buffer (10 mM sodium phosphate, 1 mM TCEP, pH 6.9) was measured in a 1 mm quartz cuvette. The final spectra were an average of 20 scans.

## Results

### Zn^2+^ Enhances LLPS of TIA-1, Especially in the Presence of Nucleic Acid

**FIGURE 1:**
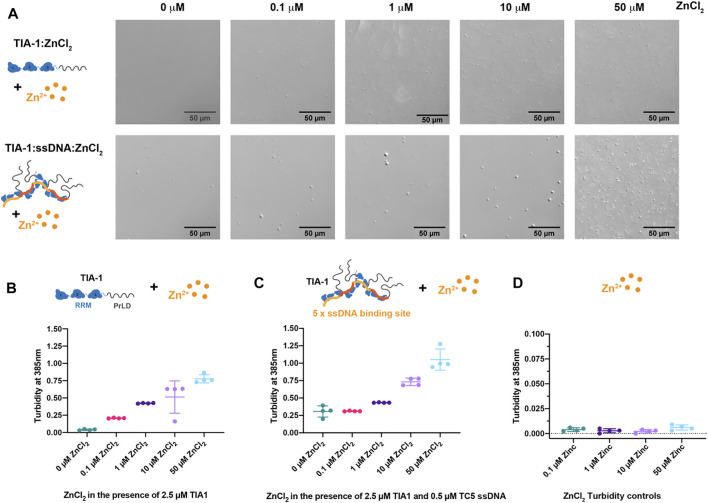
Zinc enhances LLPS of TIA-1 and TIA-1:ssDNA in a concentration dependant manner. **(A)** DIC microscopy of 2.5 μM TIA-1 (upper) and 0.5 μM ssDNA TC5 + 2.5 μM TIA-1 (lower) in the presence of 0–50 μM ZnCl_2_. **(B)** Turbidity (A_385 nm_) of 2.5 μM TIA-1 + 0–50 μM ZnCl_2_. **(C)** Turbidity of 2.5 μM TIA-1 + 0.5 μM ssDNA TC5 + 0–50 μM ZnCl_2_ and **(D)** Turbidity of 0–50 μM ZnCl_2_ alone. Conditions: 20 mM HEPES, 50 mM NaCl, 12 mM Arginine, pH 7.2, at 25°C and within 20 min of incubation. 4 replicates. Error bars represent S.D.

To explore the effect of Zn^2+^ on TIA-1 LLPS and potential subsequent aggregation, we first assessed the effect of Zn^2+^ on the LLPS of TIA-1 protein in the absence of nucleic acid. In this study, purified monomeric TIA-1 protein was prepared in the presence of 500 mM arginine, then diluted into a low salt buffer to a concentration just below its saturation (*Csat*), at which TIA-1 shows minimal LLPS (Loughlin et al., 2021). The effect of ZnCl_2_ on the LLPS of TIA-1 protein was analysed by DIC microscopy and quantified using turbidity (absorbance at 385 nm). TIA-1 protein alone at 2.5 μM showed minimal LLPS when imaged by DIC (Figure 1A). Addition of 0.1–50 μM ZnCl_2_ resulted in the appearance of spherical droplets that escalated in number on increasing Zn^2+^ concentration, demonstrating ZnCl_2_ enhanced LLPS of TIA-1 (Figure 1A). The turbidity of TIA-1 also increased in the presence of ZnCl_2_ in a concentration-dependent manner from 0.2 to 0.8, reflecting increased LLPS (Figure 1B). Taken together, these results show that ZnCl_2_ alone enhances LLPS of TIA-1, in agreement with previous studies of TIA-1 fusion protein (Rayman et al., 2018).

Our previous work showed that nucleic acid harbouring multiple binding sites enhances LLPS of TIA-1, lowering the concentration at which the protein spontaneously phase separates (Loughlin et al., 2021). To determine whether nucleic acid and Zn^2+^ enhance LLPS of TIA-1 cooperatively or act in a competitive manner, we analysed the effect of ZnCl_2_ on ssDNA-induced LLPS of TIA-1. TIA-1 (at 2.5 μM) in the presence of ssDNA harbouring five TIA-1 binding sites (“TC5”) was assessed for LLPS using DIC microscopy and quantified by turbidity. DIC showed the presence of LLPS droplets (∼2–5 μm in diameter) that were slightly larger than those instigated only by ZnCl_2_ and gave rise to turbidity measurements of ∼0.3 (Figures 1A,C). Addition of 0.1 μM ZnCl_2_ had minimal effect on the turbidity of TIA-1:ssDNA; however, 1–10 μM ZnCl_2_ enhanced the number of droplets and increased turbidity to 0.4-0.75, showing that Zn^2+^ and ssDNA together enhance LLPS of TIA-1. Addition of 50 μM ZnCl_2_ resulted in a further increase in turbidity to ∼1.0, and DIC showed the presence of a large number of smaller droplets, suggesting that at these concentrations Zn^2+^-induced TIA-1 LLPS dominates the phase separation of these samples. It was also confirmed that ZnCl_2_ alone did not give rise to sample turbidity (Figure 1D). These results show that Zn^2+^ further enhances nucleic acid-induced LLPS of TIA-1.

### Zn^2+^ Interacts Specifically With TIA-1 RRM2 Residues

**FIGURE 2:**
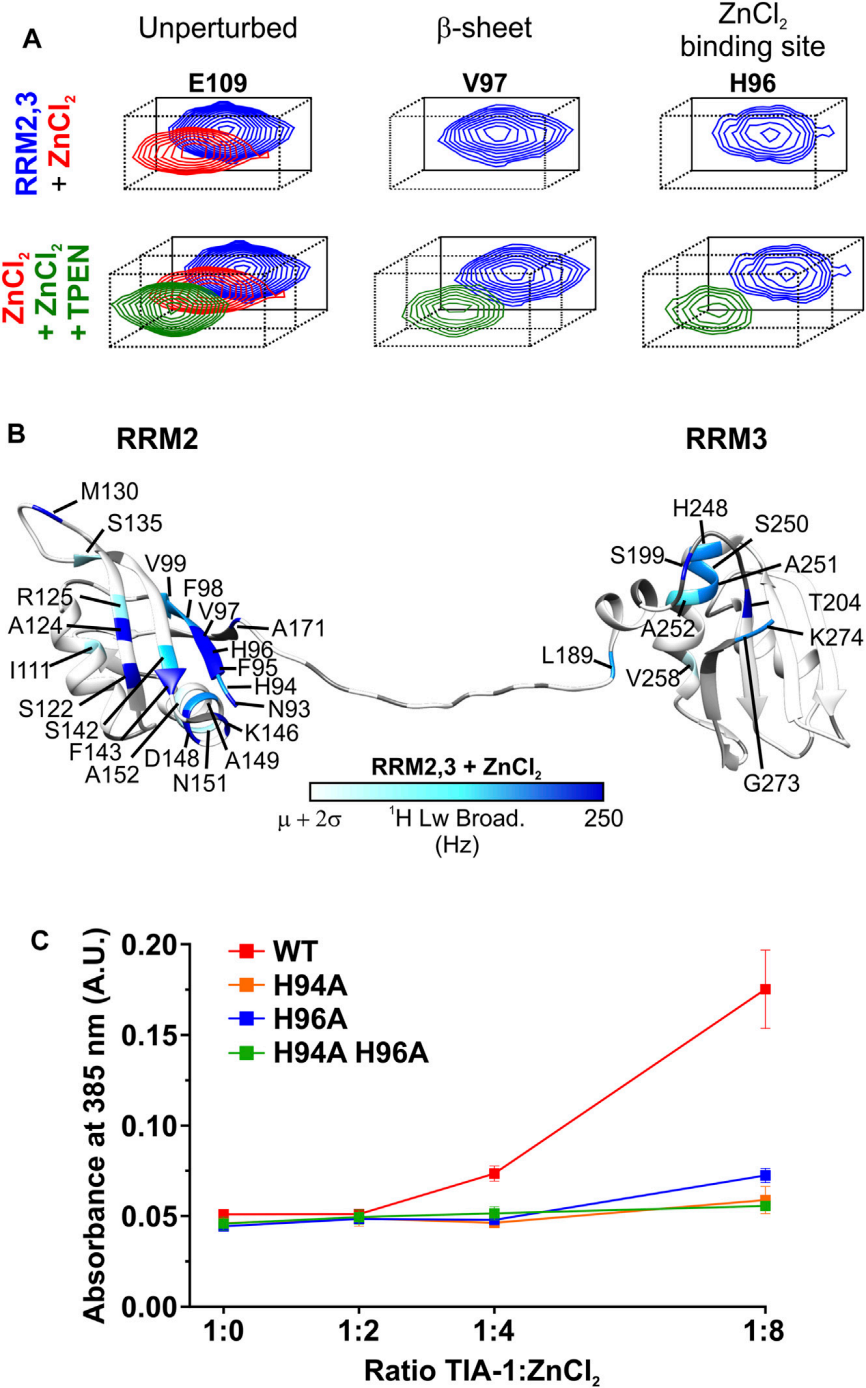
Zn2+ specifically binds to TIA-1 RRM2,3 and mediates protein multimerization. **(A)** Detailed view of representative amide resonances of unperturbed (*left panel*), *ß*-sheet (*middle panel*) or Zn^2+^ binding site (*left panel*) TIA-1 RRM2,3 residues from the superimposed [^1^H-^15^N] HSQC spectra of ^15^N-labeled TIA-1 RRM2,3 construct either free (*blue*) or upon incubation with ZnCl_2_ (*red*) or ZnCl_2_ + TPEN (*dark green*). Whole views of 2D NMR spectra are included in Supplementary Figures S2A,E. **(B)** Map of ^1^H-linewidths on the TIA-1 RRM2,3 ribbon upon addition of an equimolar ratio of ZnCl_2_. Residues with ^1^H line broadening larger than the average (*µ*) plus two standard deviations (*σ*) are colored using a *white-to-blue* scale. Prolines and unassigned residues are colored in grey. TIA-1 PDB ID: 2MJN (Wang et al., 2014). Conditions: 20 mM HEPES, 50 mM NaCl, 5 mM TCEP, pH 6.9, at 25°C and upon 30 min incubation with ZnCl_2_ or ZnCl_2_ +TPEN. **(C)** Comparison of turbidity (A_385 nm_) produced by TIA-1 RRM2,3 WT and different His-to-Ala mutants at increasing concentrations of ZnCl_2_. All measurements were performed by using TIA-1 constructs at 20 µM concentration. Conditions: 20 mM HEPES, 50 mM NaCl, 1 mM TCEP, pH 6.9, at 25°C and within 30 min incubation under shaking. 3 replicates. Error bars represent S.D.

In order to explore the molecular basis for Zn^2+^-induced LLPS of TIA-1, NMR ^1^H-^15^N-HSQC titration experiments were undertaken for RRM1 (amino acids 1-81) and RRM2,3 (amino acids 93-274) domains. ZnCl_2_ was titrated into ^15^N-labelled RRM1 or RRM2,3 at 200 and 300 μM, respectively, at pH 6.9. Addition of Zn^2+^ into either sample resulted in some turbidity observed by eye, suggesting a decrease in solubility at these concentrations. The RRM1 amide resonances showed no significant changes in either chemical-shift perturbations or linewidths; therefore, no specific Zn^2+^ binding site was identified (Supplementary Figure S1). In contrast, when Zn^2+^ was added to a sample of RRM2,3, the average broadening was enhanced from 25.5 ± 4.8 Hz (free TIA-1 RRM2,3) to 37.4 ± 18.3 Hz (Zn^2+^:protein ratio of 1:1), indicative of multimerization of TIA-1 (Supplementary Figure S2). Furthermore, our analysis also revealed a subset of amide resonances undergoing specific line broadening (>250 Hz). Interestingly, some of the affected residues—namely N93 (*ß*_1_), F95 (*ß*_1_), H96 (*ß*_1_), V97 (*ß*_1_), S122 (*ß*_2_), A124 (*ß*_2_) and F143 (*ß*_3_)—are positioned in a cluster adjacent to and including RRM2 *ß*-sheet residues of the canonical RNA binding site (Figures 2A,B and Supplementary Figure S2A). Moreover, other residues at RRM2 α_2_-helix (K146 and D148) and those located at a flexible RRM2 loop and the interdomain linker (M130 and A171, respectively) were also substantially broadened (>250 Hz; Figures 2A,B). Moderate, but still significant, broadening (> mean + 2σ Hz) was also observed for resonances H94 (*ß*_1_), F98 (*ß*_1_), V99 (*ß*_1_), I111 (α_1_), R125 (*ß*_2_), S135 (*ß*_3_), S142 (*ß*_3_), A149 (α_2_), N151 (α_2_) and A152 (α_2_) in RRM2 (Figures 2A,B, Supplementary Figure S2). Several residues from the linker and RRM3 also broadened above the threshold, but not to the extent of that observed in RRM2. Remarkably, both substantial line broadening of RRM2 in RRM2,3 and sample turbidity were fully reversible upon addition of the Zn^2+^-chelator TPEN (average linewidth of 26 ± 4 Hz), which confirms that these effects are unequivocally induced by Zn^2+^ (Figure 2A, Supplementary Figures S2E,G). It was also confirmed that TPEN alone had no effect on TIA-1 RRM2,3 resonances (Supplementary Figures S2F,G). Together, these results suggest that Zn^2+^ mediates the multimerization of TIA-1 RRM2,3 domains independently of the PrLD at high protein concentration (300 µM).

From these data, H94 and H96 in RRM2 stand out as potential Zn^2+^ ligands of TIA-1 as histidine residues are often involved in Zn^2+^ coordination (Kocyla et al., 2021). Based on this premise, the NMR titration was repeated under lower pH conditions at which histidine residues could not interact with Zn^2+^ due to protonation (Cruz-Gallardo et al., 2013; Cruz-Gallardo et al., 2015). Titrating TIA-1 RRM2,3 with Zn^2+^ at pH 5.5 resulted in neither enhanced line broadening nor visible turbidity, consistent with the lack of histidine coordination to Zn^2+^ in TIA-1 RRM2,3 (Supplementary Figures S2B–D). It should be noted that Zn^2+^ addition to a solution of TIA-1 RRM1 at pH 5.5 also did not produce any visible turbidity and the linewidth of the signals remained unaltered (Supplementary Figures S1B–D). Thus, histidines in RRM1, that occur at positions 54, 56 and 58, could also transiently coordinate Zn^2+^, but without forming stable complexes. Altogether, NMR titrations identified TIA-1 RRM2 H94 and H96 residues as potential candidates for specific Zn^2+^ binding, possibly mediating the formation of Zn^2+^-induced multimers underlying the observed solution turbidity for TIA-1 RRM2,3.

To test this hypothesis we first quantified the effect of Zn^2+^ titration into 20 µM TIA-1 RRM2,3 on solution turbidity—as measured by absorbance at 385 nm. Increasing the Zn^2+^ enhanced the turbidity of RRM2,3 in a concentration dependent manner (Supplementary Figure S3A). Furthermore, under these conditions, the suppressing effects of TPEN and pH 5.5 in reducing TIA-1 RRM2,3 turbidity were also observed, as previously seen in the more concentrated TIA-1 RRM2,3 NMR solutions (Supplementary Figures S3B,C). Thus, in order to confirm the role of H94 and H96 in Zn^2+^-induced multimerization, TIA-1 RRM2,3 mutants were designed so that one or both histidine residues were substituted by alanine. The correct protein folding was confirmed using CD spectropolarimetry, revealing the same secondary structure content for TIA-1 RRM2,3 wild type and mutants (Supplementary Figure S4). While addition of Zn^2+^ to a ratio of 8:1 increased the turbidity of TIA-1 RRM2,3 (20 µM), minimal or negligible effects were observed for H94A, H96A or H94A H96A mutants (Figure 2C). This finding suggests a structural role for Zn^2+^ in specifically coordinating H94/H96 residues in RRM2, underlying Zn^2+^-induced multimerization of TIA-1 RRMs.

### Zn^2+^ Suppresses Nucleic Acid Induced TIA-1 Fibrillar Aggregation

**FIGURE 3:**
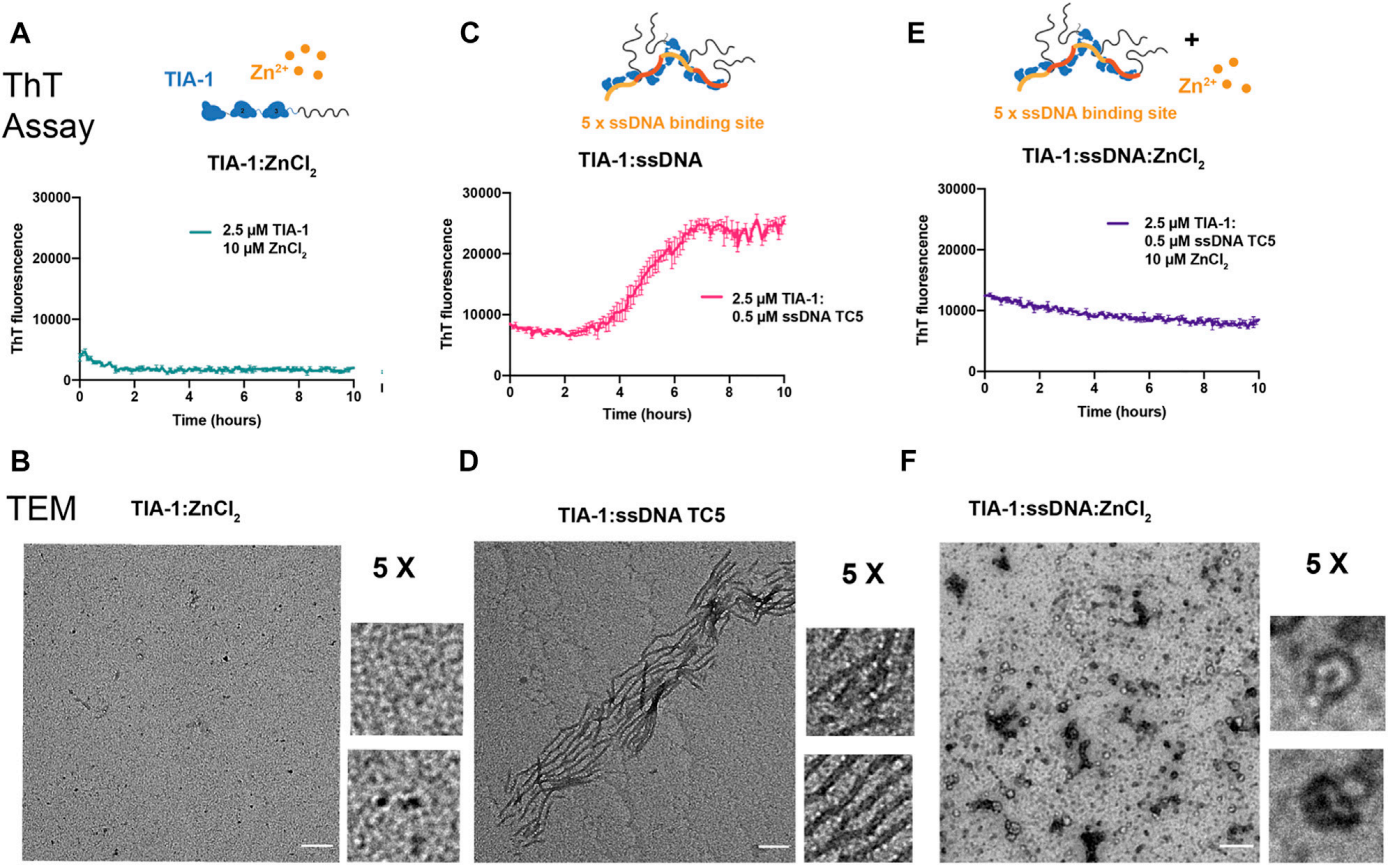
Zinc inhibits fibrillar aggregation of TIA-1. Thioflavin T (ThT) fluorescence assay monitoring the formation of ThT positive aggregates (*top*) and TEM of final samples (*bottom*) **(A,B)** 2.5 μM TIA-1, 10 μM ZnCl_2_ **(C,D)** 2.5 μM TIA-1, 0.5 μM ssDNA TC5 **(E,F)** 2.5 μM TIA-1, 0.5 μM ssDNA TC5, 10 μM ZnCl_2_. Conditions: 20 mM HEPES, 50 mM NaCl, 15 mM Arginine, pH 7.2, at 30°C with shaking. 3 replicates. Errors represent S.D. Scale bar represents 200 nm.

Whilst LLPS of TIA-1 contributes to stress granule formation, aberrant LLPS of disease-associated TIA-1 variants and enhanced propensity to form fibrillar aggregates are associated with impaired stress granule dynamics facilitating pathological inclusions linked to ALS (Mackenzie et al., 2017; Zhang et al., 2019). Thus, we next probed the effect of Zn^2+^ on fibrillar aggregation of TIA-1 over time, in and out of the presence of nucleic acid, as monitored by Thioflavin T (ThT) fluorescence and TEM. At concentrations at which TIA-1 shows minimal LLPS (2.5 μM), no significant TIA-1 aggregation was detected (Supplementary Figure S5A). Addition of 10 μM ZnCl_2_, that robustly induces LLPS of TIA-1, did not enhance aggregation of TIA-1 and even appeared to show a slight suppression of ThT fluorescence (Figure 3A). TEM measurements did not detect any fibrillar aggregation (Figure 3B), showing that Zn^2+^-induced LLPS of TIA-1 does not produce fibrillar aggregates.

We then undertook the same experiment in the presence of the TC-rich dsDNA harbouring 5 TIA-1 binding sites (“TC5”), that enhances TIA-1 LLPS and fibril formation (Loughlin et al., 2021). As previously observed, addition of TC5 to TIA-1 (2.5 μM) resulted in fibrillar aggregation as detected by ThT fluorescence over time, and fibrils as observed by TEM (Figures 3C,D). To test the effect of Zn^2+^ on nucleic acid-induced fibrilization of TIA-1, we monitored aggregation of TIA-1 in the presence of TC5 and 10 μM ZnCl_2_. No enhancement in ThT fluorescence was observed over time and TEM of final samples did not show numerous fibrils, but small aggregates with potential oligomeric species (Figures 3E,F). Interestingly, we also observed inhibition of ThT fluorescence at substoichiometric concentrations of ZnCl_2_ (Supplementary Figure 5B). These results suggest that the presence of Zn^2+^ impedes fibrilization of TIA-1, maintaining it within a reversible LLPS state.

### RGG-Rich RBD of FUS Enhances Nucleic Acid Induced TIA-1 LLPS and Prevents Maturation to Irreversible Aggregates

**FIGURE 4:**
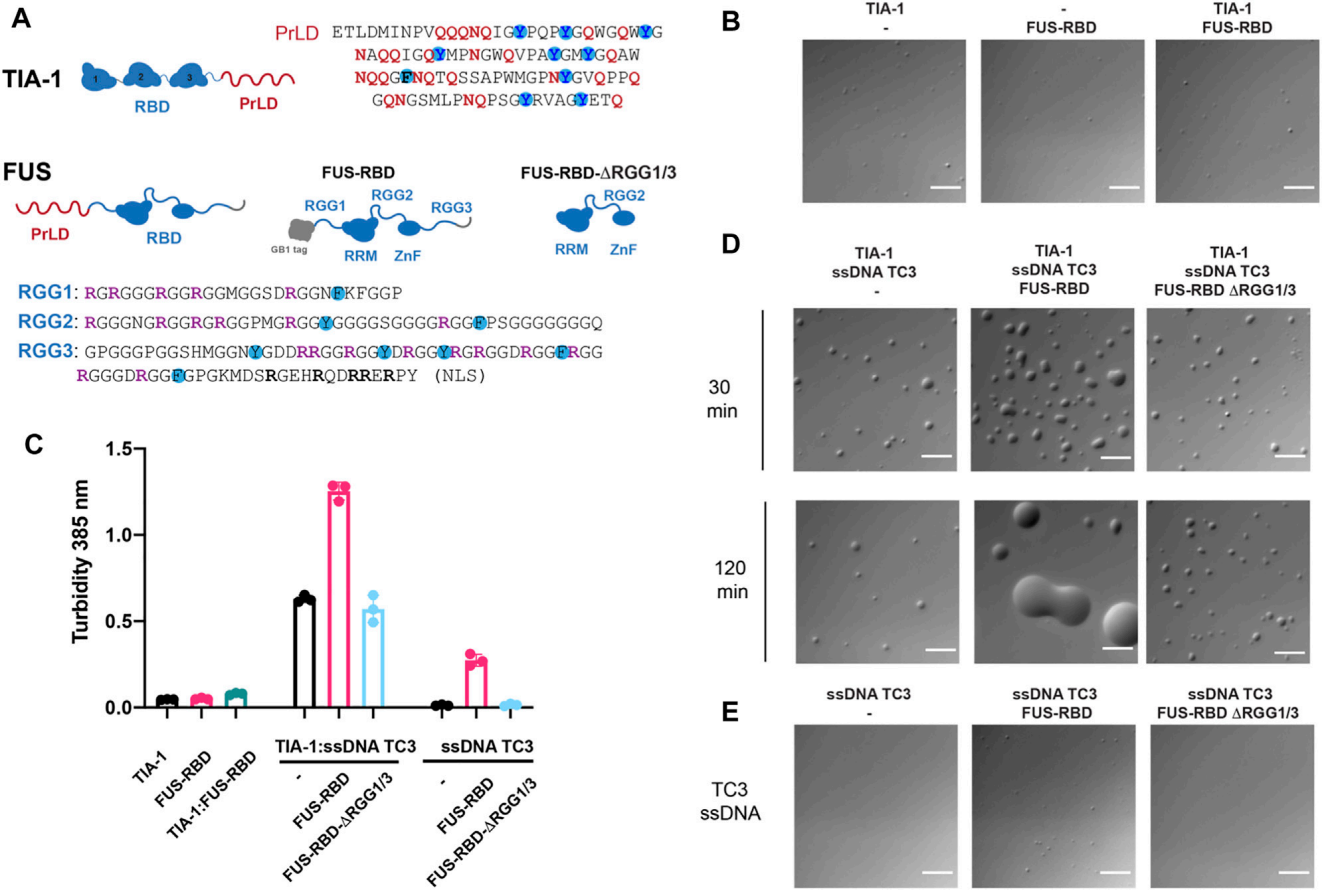
FUS-RBD enhances nucleic acid induced LLPS of TIA-1. **(A)** Domain structures of TIA-1 (with PrLD sequence alongside highlighting Q, N and Y amino acids) and FUS constructs: FUS-RBD and FUS-RBD-ΔRGG1/3 constructs (with RGG sequences shown underneath highlighting R, F and Y amino acids). **(B)** DIC microscopy of 2.5 μM TIA-1; 0.5 μM ssDNA TC3; 2.5 μM FUS-RBD-RGG; 2.5 μM FUS-RBD-short at room temperature **(C)** Turbidity assay of 2.5 μM TIA-1; 0.5 μM ssDNA TC3; 2.5 μM FUS-RBD-RGG; 2.5 μM FUS-RBD-short. 20 min incubation at 25°C. 3 replicates. Error bars represent S.D. **(D)** DIC microscopy of 2.5 μM TIA-1 combined with 0.5 μM ssDNA TC3 alone or with 2.5 μM FUS-RBD-RGG or 2.5 μM FUS-RBD-short at 30 and 120 min incubation at room temperature. **(E)** 0.5 μM ssDNA TC3 alone or with 2.5 μM FUS-RBD-RGG or 2.5 μM FUS-RBD-short. Scale bars depict 10 μm. Conditions: 20 mM HEPES, 50 mM NaCl, 12 mM arginine, pH 7.2, at 25°C.

As part of our study of stress granule co-factors, we also analysed the effects of the RGG-rich RNA-binding domain (RBD) of FUS—as a representative RGG-rich protein and potential molecular modulator of TIA-1 LLPS. FUS localises to stress granules *via* its RGG-rich intrinsically disordered region RGG3 (Bentmann et al., 2012). Moreover, *in vitro* TIA-1 protein partitions into phase separated FUS protein mediated in part by the RGG rich RBD (Wang et al., 2018). Interestingly, although RGG IDPs can drive homotypic LLPS, unlike PrLD-mediated LLPS, they do not readily mature into irreversible fibrillar aggregates (Gui et al., 2019). Thus, interactions of RGG intrinsically disordered regions may also influence TIA-1 LLPS and potential fibrillar aggregation. We therefore tested the effect of RGG-rich RBD of FUS on TIA-1 LLPS and aggregation, by DIC and turbidity measurements, both alone and in the presence of nucleic acids. FUS-RBD (amino acids 242-526) comprising RGG1-RRM-RGG2-ZnF-RGG3 (Figure 4A) was purified in high salt to homogeneity, free from RNA contamination. TIA-1 and FUS-RBD diluted to 2.5 µM in aggregation buffer showed minimal LLPS in DIC microscopy, with no apparent increase upon their addition (Figure 4B), and only a very small increase in turbidity was observed upon their addition (Figure 4C). Together, this suggests that FUS-RBD does not greatly enhance LLPS of TIA-1 at these concentrations.

Next, we tested the effect of FUS-RBD on TIA-1 LLPS in the presence of a ssDNA comprising three tandem TIA-1 binding sites (“TC3”). The addition of sub-stoichiometric amounts of TC3 to TIA-1 dramatically enhanced LLPS, as measured by increased solution turbidity (Figure 4C) and by droplets observed by DIC that were stable over 2 h (Figure 4D). Addition of equimolar FUS-RBD to TIA-1 and TC3 further enhanced LLPS, resulting in greatly increased turbidity and substantially larger LLPS droplets (Figures 4C,D). Unlike the stable TIA-1:TC3 samples, TIA-1:TC3:FUS-RBD condensates fused together over 2 h. To test whether these changes were due to RGG regions of FUS, a short version of FUS RBD (amino acids 269-454) without RGG1 or RGG3 regions (FUS-RBD-ΔRGG1/3) was mixed with TIA-1:TC3. On addition of FUS-RBD-ΔRGG1/3, no change in turbidity or appearance of LLPS was observed, with droplets appearing relatively stable over a 2 h time period (Figures 4C,D). Although TC3 was designed with TIA-1 binding sites, addition of TC3 ssDNA to FUS-RBD resulted in increased turbidity, with a small increase in the number of LLPS droplets (Figures 4C,E). In contrast, the addition of TC3 to FUS-RBD-ΔRGG1/3 did not increase turbidity or droplet formation (Figures 4C,E). In order to check whether FUS-RBD could be forming promiscuous interactions by displacing TIA-1 from the TC3 ssDNA, we determined its direct binding affinity to a TC DNA sequence in comparison to that of TIA-1 RRM2,3. This showed that the FUS-RBD affinity (*K*_d_ = 30 nM) was significantly lower than that of TIA-1 RRM2,3 (*K*_d_ = 2 nM) (Supplementary Figure S6) and is unlikely to displace TIA-1 under the conditions of the LLPS assay. These results show that the addition of an RGG-rich protein can modulate the nucleic acid-induced LLPS of TIA-1, potentially *via* interactions with RRM-bound nucleic acid and/or with TIA-1 PrLD.

**FIGURE 5:**
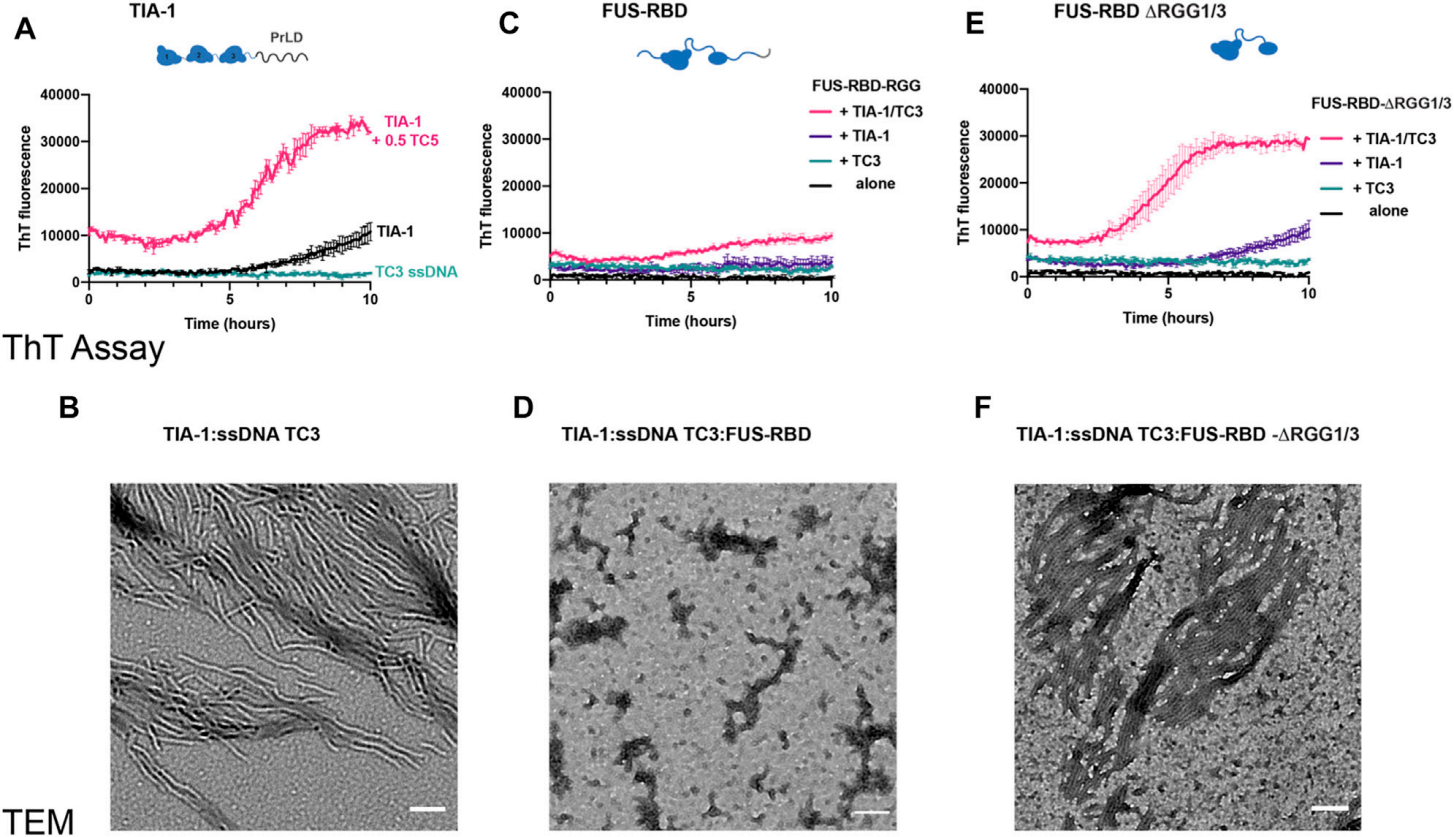
FUS RBD inhibits nucleic acid induced TIA-1 fibril formation *via* RGG IDRs **(A)** Thioflavin T (ThT) fluorescence assay monitoring fibril formation of 2.5 μM TIA-1 alone or in presence of 0.5 μM ssDNA TC3. **(B)** TEM of 2.5 μM TIA-1, 0.5 μM ssDNA TC3. **(C)** ThT fluorescence assay of 2.5 μM FUS-RBD-RGG alone and in combinations with 2.5 μM TIA-1, 0.5 μM ssDNA TC3. **(D)** TEM 2.5 μM TIA-1, 0.5 μM ssDNA TC3, 2.5 μM FUS-RBD-RGG. **(E)** ThT fluorescence assay of 2.5 μM FUS-RBD-short alone and in combinations with 2.5 μM TIA-1, 0.5 μM ssDNA TC3. **(F)** TEM of 2.5 μM TIA-1, 0.5 μM ssDNA TC3, 2.5 μM FUS-RBD-short. Conditions: 20 mM HEPES, 50 mM NaCl, 15 mM arginine, pH 7.2, at 30°C with shaking. 3 replicates. Errors represent S.D.

Following the observation of changes in LLPS of TIA-1:TC3 condensates, we next examined the fibril formation capacity of TIA-1 in the presence of RGG-rich FUS-RBD as monitored by ThT fluorescence and TEM. Whereas 2.5 µM TIA-1 showed minimal aggregation with only a small increase in ThT fluorescence after ∼8 h, TC3 ssDNA efficiently enhanced ThT fluorescence over time, resulting in amyloid-like fibrils, as observed under TEM (Figures 5A,B). In contrast, the addition of FUS-RBD to TIA-1:TC3 resulted in a dramatic reduction in ThT fluorescence, suggesting a substantial decrease in fibril formation, confirmed by TEM imaging (Figures 5C,D). Interestingly, the small increase in ThT fluorescence of TIA-1 alone is also inhibited in the presence of FUS-RBD (Figure 5C). To assess whether suppression of TIA-1 fibril formation was due to RGG repeats of FUS-RBD, we tested TIA-1:TC3 aggregation in the presence of FUS-RBD-ΔRGG1/3. FUS-RBD-ΔRGG1/3 did not suppress TIA-1:TC3 fibril formation, with a sigmoidal increase in ThT fluorescence occurring and amyloid-like fibrils observed in TEM (Figures 5E,F). Similarly, FUS-RBD-ΔRGG1/3 did not suppress the small increase in ThT fluorescence of TIA-1 alone. Finally, neither FUS-RBD nor FUS-RBD-ΔRGG1/3, alone or in the presence of TC3, showed evidence of fibril formation (Figures 5C,E). These results indicate that RGG intrinsically disordered domains of FUS-RBD prevent the fibril formation of TIA-1 under these conditions.

## Discussion

Stress granules are transient in nature. Both internal and temporal dynamics are important for stress granule function. Decreased stress granule dynamics or delayed disassembly can result in the pathological aggregates observed in neurodegenerative diseases. TIA-1 is an aggregation-prone RNA-binding protein that contributes to stress granule formation. Within stress granules, TIA-1 protein is mobile (Tauber et al., 2020); however, it also has an inherent propensity to form irreversible aggregates (Gilks et al., 2004), linked to disease (Mackenzie et al., 2017). This study set out to investigate the way in which stress granule components influence LLPS and fibrillar aggregation of TIA-1 *in vitro*. In this work, we have shown that two stress granule substituents, Zn^2+^ and a representative RGG-rich domain from FUS, each enhance nucleic acid-induced LLPS of TIA-1, yet inhibit formation of irreversible fibrils *in vitro.* Furthermore, we have identified a Zn^2+^-binding site involving two histidine residues in RRM2 that appear to mediate multimerization of TIA-1 RRMs independently of the PrLD. Our studies thus exemplify the role of heterotypic interactions in the maintenance of biomolecular condensates and prevention of their progression to irreversible aggregates (Alberti and Hyman, 2021).

Many RNA-binding proteins that possess both RBDs and low complexity PrLDs are prone to aggregation *in vitro* (Molliex et al., 2015; Patel et al., 2015; Mackenzie et al., 2017). Yet, in the cellular environment these proteins remain largely soluble due to regulation, for example, by extensive post-translational modifications (Hofweber and Dormann, 2019; Velázquez-Cruz et al., 2021). Changes in propensity for LLPS and aggregation have been investigated *in vitro* in response to phosphorylation of tyrosine residues in the PrLD (Kato et al., 2012; Monahan et al., 2017), methylation of arginine residues (Nott et al., 2015), intramolecular interactions with other protein domains (Martin et al., 2021) as well as RNA abundance (Maharana et al., 2018; Alshareedah et al., 2019). Notably, many of these modifications have the same effect on both LLPS and irreversible aggregation. In contrast, this work demonstrated two heterotypic interactions that enhance LLPS, but inhibit fibrillar aggregation and, as such, may provide a glimpse of the way in which the cell could harness heterotypic interactions to inhibit the aggregation potential of stress granule proteins*.*

Zn^2+^ has been proposed as a co-factor for TIA-1, promoting self-multimerization, LLPS and localisation to stress granules under conditions of cellular stress (Rayman et al., 2018). In agreement with Rayman et al., we showed that Zn^2+^ induces LLPS of TIA-1 *in vitro.* In addition, we showed that Zn^2+^ significantly enhances nucleic acid-induced LLPS. It is possible that an increase in cytosolic Zn^+^ and the availability of exposed RNA-binding sites due to stalled polysomes, could work together to contribute to the rapid response of TIA-1 to stress in cells. In fact, cellular studies revealed that while Zn^2+^ was insufficient to trigger the formation of TIA-1 positive stress granules, it potentiated the effects of arsenite stress to do so (Rayman et al., 2018). It should be noted that our experiments, including those demonstrating Zn^2+^ induced multimerization of TIA-1 RRM2,3, utilized low to mid-micromolar concentrations of Zn^2+^. While total zinc concentrations in cells have been measured to be 200–300 μM, free Zn^2+^ concentrations are estimated to be in the low nanomolar to picomolar range (Maret, 2015). Thus, consistent with the proposed mechanism of Zn^2+^ promoted stress granule formation, normal cellular concentrations of Zn^2+^ would not be expected to induce TIA-1 phase separation. But upon cellular stress, as shown by Rayman et al., free Zn^2+^ in the cell was detected at levels consistent with the presence of low micromolar concentrations of free Zn^2+^ (Rayman et al., 2018). This may be sufficient, depending upon localised concentrations of TIA-1 and Zn^2+^ in the cell, for Zn^2+^ to assist TIA-1 multimerization *via* histidine chelation in the process of phase separation. It will be of great interest to verify the importance of histidines in cellular studies of TIA-1 histidine mutants.

This study also revealed that the molecular mechanisms of Zn^2+^-induced LLPS of TIA-1 may involve specific Zn^2+^ interactions with its structured RRM2 domain. Indeed, we identified H94 and H96 as specific Zn^2+^-binding sites in the RRM2 of TIA-1 that mediated multimerization of the tandem RRM2,3 domains. Interestingly, these residues would most likely link two TIA-1 molecules *via* tetrahedral coordination of a Zn^2+^ ion leading to dimer formation. This would not lead to multimerization on its own and thus implies that another mode of interaction also can occur between TIA-1 RRM2,3 molecules. In the context of full-length TIA-1, this suggests that Zn^2+^ may contribute to LLPS of TIA-1 by promoting inter-RRM2 interactions in addition to the self-association of PrLD domains. The role of Zn^2+^ in promoting protein-protein association *via* specific contact sites is well documented (Kocyla et al., 2021), but there are less examples of specific Zn^2+^ interactions promoting LLPS. Zn^2+^ has been shown to enhance LLPS of the disordered Tau protein through multiple distinct binding sites (Singh et al., 2020) as well as to assist the aggregation of SPQF through specific sites in adjacent coiled coils (Huang et al., 2020). Yet to our knowledge, Zn^2+^-mediated multimerization of globular RRM domains would represent an unusual molecular interaction contributing to functional LLPS.

A striking observation from our study was the blockade of TIA-1 aggregation by Zn^2+^ in the presence of a multisite nucleic acid that would have otherwise induced amyloid-like fibril formation. Even sub-stoichiometric concentrations of Zn^2+^ were sufficient to inhibit nucleic acid-induced fibrilization of TIA-1. Elevated levels of Zn^2+^ detected under conditions of cellular stress could act to prevent aberrant TIA-1 aggregation within stress granules, facilitating normal stress granule dynamics critical for its function. Such an inhibitory effect at low Zn^2+^ concentrations may be due to transient interactions with residues in the PrLD, a mechanism that has been proposed for the Zn^2+^-mediated inhibition of Aβ amyloid fibril formation (Matheou et al., 2016). Due to solubility limits, we were unable to directly test Zn^2+^ binding to the full PrLD regions of TIA-1 using NMR spectroscopy. However, glutamine and asparagine residues, frequent in PrLDs, stand out as potential Zn^2+^ interaction sites. Alternatively, the same Zn^2+^ interactions that promote TIA-1 RRM2,3 multimerization could also act to interfere with the formation of fibrillar aggregates. Zn^2+^ may enhance TIA-1 RRM interactions resulting in orientations between TIA-1 molecules that hinder PrLD propagation to fibrils. Instead, a different network of interactions may be favoured. Interestingly, a number of spherical particles reminiscent of oligomers were observed in TEM images of TIA-1:ssDNA:Zn^2+^condensates, similar to those observed in mouse TIA-1 in a colloidal dense phase (Fritzsching et al., 2020). It will be interesting to see whether Zn^2+^ is either broadly protective against PrLD fibrillar aggregation in other RNA binding proteins with PrLDs or specifically protective to TIA-1. By either mechanism, our study suggests that Zn^2+^ may be highly important to the healthy maintenance of dynamic stress granules.

A remarkable inhibition of irreversible fibrillar aggregation of TIA-1:nucleic acid was also observed in the presence of the RGG-rich FUS-RBD. It is already understood that low complexity regions rich in Arg and Gly amino acids (RGG) can enhance LLPS through RNA binding, homotypic interactions with other RGG regions, or heterotypic interactions with PrLDs (Qamar et al., 2018; Kaur et al., 2021). Here we showed that addition of FUS-RBD further enhances LLPS of nucleic acid-bound TIA-1, but suppresses fibril formation over time through RGG-rich regions. This is most likely to be due to direct interference of TIA-1 PrLD homotypic interactions that underlie fibrillar aggregation of TIA-1. PrLDs drive LLPS of RNA binding proteins *via* multivalent contacts dominated by π-π interactions between Tyr residues (Martin et al., 2020). RGG-rich domains, however, can directly compete with these interactions though heterotypic π-cation interactions between Tyr and Arg residues, as has been observed in LLPS of FUS (Wang et al., 2018). This same type of interaction is most likely also the basis for the maintenance of soluble full-length TIA-1 in the presence of arginine in the current study. These results suggest that heterotypic interactions between RGG and PrLD of different RNA-binding proteins could contribute to maintaining the dynamic and liquid state of LLPS *in vitro*, referred to as heterotypic buffering (Mathieu et al., 2020). Stress granules contain hundreds of proteins, including an over representation of RNA binding proteins with PrLDs such as TIA-1 and hnRNPA1, and RGGs such as in the major stress granule protein G3BP. Interaction between RGG and PrLD low complexity regions may contribute to maintaining the dynamic nature of stress granules, vital for their function.

Together, these *in vitro* studies suggest mechanisms through which TIA-1 LLPS is enhanced and aggregation is inhibited. While Zn^2+^ interactions with TIA-1 RRM2 may promote multimerization, heterotypic interactions of Zn^2+^ and RNA-binding proteins rich in RGG repeats may prevent maturation of TIA-1 from reversible liquid condensates to amyloid fibrils. These mechanisms likely occur in parallel with other molecular modifications and interactions within stress granules to protect against harmful aggregation.

## Data Availability

The original contributions presented in the study are included in the article/Supplementary Material, further inquiries can be directed to the corresponding authors.
