# Supplementary material for: Regulation of TIA-1 Condensates: Zn2+ and RGG Motifs Promote Nucleic Acid Driven LLPS and Inhibit Irreversible Aggregation
Source: Front Mol Biosci. 2022 Jul 14;9:960806. doi: 10.3389/fmolb.2022.960806 (PMC9329571; doi:10.3389/fmolb.2022.960806)
Supplement: Supplementary file 1 [file DataSheet1.PDF]

Supplementary Material

Regulation of TIA-1 condensates: Zn^{2+} and RGG motifs promote nucleic acid driven LLPS and inhibit irreversible aggregation

Danella L. West, Fionna E. Loughlin, Francisco Rivero-Rodriguez, Naveen Vankadari, Alejandro Velázquez-Cruz, Laura Corrales-Guerrero, Irene Díaz-Moreno and Jacqueline A. Wilce

Supplementary Figure 1. NMR titration of TIA-1 RRM1 with $ZnCl_2$ at pH 5.5 and 6.9.

Supplementary Figure 2. Effect of pH and TPEN on TIA-1 RRM2,3 in complex with $ZnCl_2$.

Supplementary Figure 3. Turbidity assays exploring the effect of TPEN and pH on TIA-1 RRM2,3 self-association.

Supplementary Figure 4. Secondary structure assessment of TIA-1 His-to-Ala mutants.

Supplementary Figure 5. Additional TIA-1 aggregation assays.

Supplementary Figure 6. Binding affinity of FUS-RBD to TC DNA.

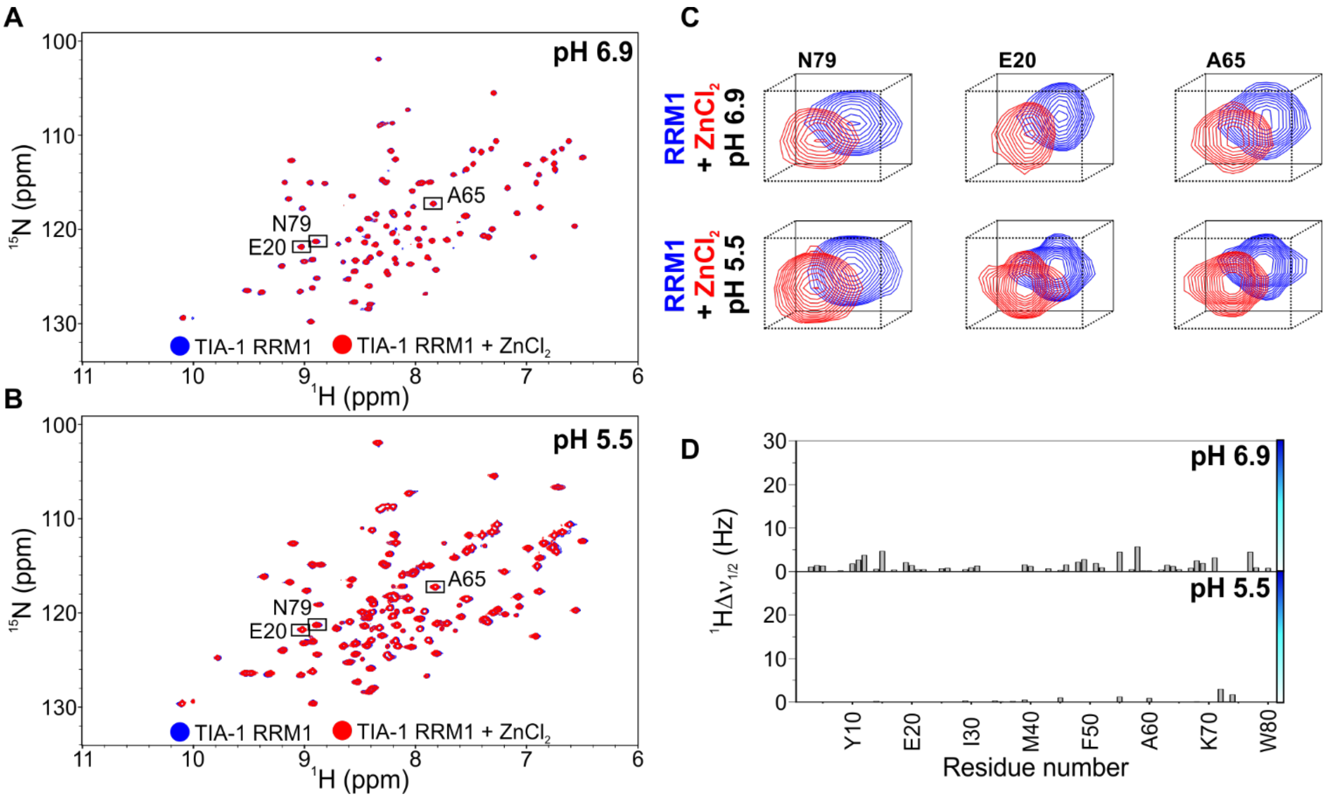

Supplementary Figure 1. NMR titration of TIA-1 RRM1 with $ZnCl_2$ at pH 5.5 and 6.9. (**A-B**) Superimposition of the [^{1}H-^{15}N] 2D HSQC spectra of free ^{15}N-labeled TIA-1 RRM1 (*blue*) and upon equimolar addition of $ZnCl_2$ (*red*) at pH 6.9 (**A**) or 5.5 (**B**). (**C**) Detailed view of the amide resonances highlighted on panels **A** and **B**. (**D**) Plot of the difference in proton linewidth (^{1}H$\Delta\nu_{1/2}$) between ^{15}N-labeled TIA-1 RRM1 upon incubation with $ZnCl_2$ at 1:1 molar ratio and free ^{15}N TIA-1 RRM1 at pH 6.9 (*upper graph*) or pH 5.5 (*lower graph*), as a function of residue number.

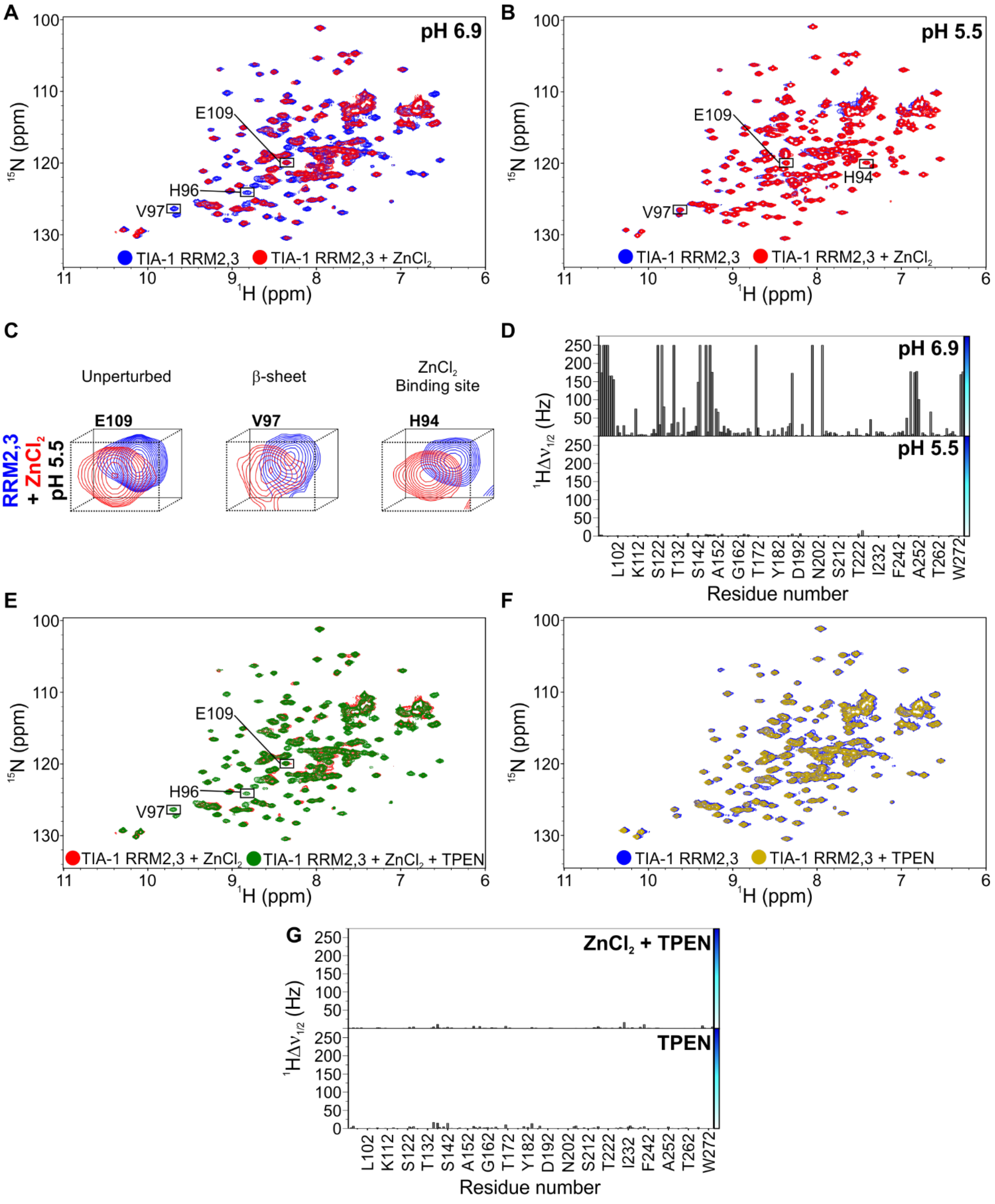

Supplementary Figure 2*.* Effect of pH and TPEN on TIA-1 RRM2,3 in complex with $ZnCl_2$. (**A**-**B**) Superimposition of the [^{1}H-^{15}N] 2D HSQC spectra of free ^{15}N-labeled TIA-1 RRM2,3 (*blue*) and upon the addition of $ZnCl_2$ at a 1:1 ratio (*red*) at pH 6.9 (**A**) or 5.5 (**B**).(**C**) Detailed

view of the amide resonances of unperturbed (*left panel*), β-sheet (*middle panel*) or $ZnCl_2$ binding site (*left panel*) residues of the 2D NMR spectra shown in panel **B**. (**D**) Plot of the difference in proton linewidth ($^1H\Delta\nu_{1/2}$) between ^{15}N TIA-1 RRM2,3 upon incubation with $ZnCl_2$ at 1:1 molar ratio and free ^{15}N TIA-1 RRM2,3 at pH 6.9 (*upper graph*) or pH 5.5 (*lower graph*) as a function of residue number. (**E**) Superimposition of the [^1H-^{15}N] 2D HSQC spectra of TIA-1 RRM2,3:$ZnCl_2$ (*red*) and TIA-1 RRM2,3:$ZnCl_2$:TPEN (*dark green*) complexes. (**F**) Superimposition of the [^1H-^{15}N] 2D HSQC spectra of free TIA-1 RRM2,3 (*blue*) and upon the addition of TPEN (*dark yellow*). (**G**) Plot of the difference in proton linewidth ($^1H\Delta\nu_{1/2}$) between ^{15}N TIA-1 RRM2,3 upon incubation with $ZnCl_2$ and TPEN (*upper graph*) or only in presence of TPEN (*lower graph*) with respect to free ^{15}N TIA-1 RRM2,3 as a function of residue number. Amide resonances included in squares in panels **A** and **E** correspond to the detailed views presented in Figure 2A of the Main Text.

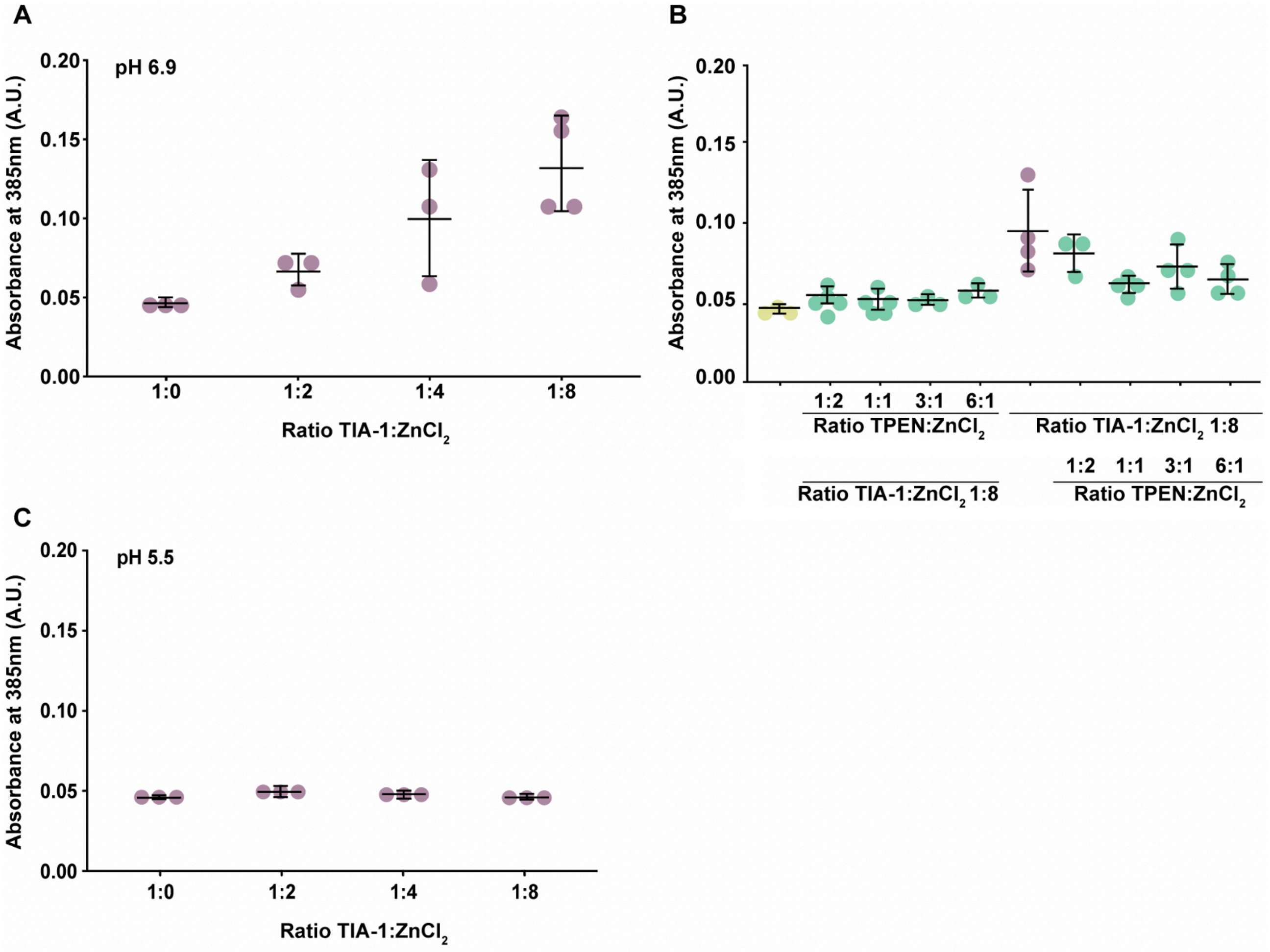

Supplementary Figure 3. Turbidity assays exploring the effect of TPEN and pH on TIA-1 RRM2,3 self-association. (**A**) Quantification of TIA-1 RRM2,3 turbidity at increasing concentrations of $ZnCl_2$. (**B**) Effect of TPEN on TIA-1 RRM2,3:$ZnCl_2$ complex formation. The left part the graph shows turbidity results obtained by incubation of TIA-1 RRM2,3 with increasing concentrations of TPEN prior to the addition of $ZnCl_2$ at a fixed 1:8 ratio (protein:$ZnCl_2$). The right part shows turbidity assays performed by first incubating TIA-1 RRM2,3 with $ZnCl_2$ at a fixed 1:8 ratio (protein:$ZnCl_2$) and adding increasing concentrations of TPEN afterwards. (C) Titration of TIA-1 RRM2,3 with increasing concentrations of $ZnCl_2$ at pH 5.5. All experiments were performed by using TIA-1 RRM2,3 at 20 μM concentration.

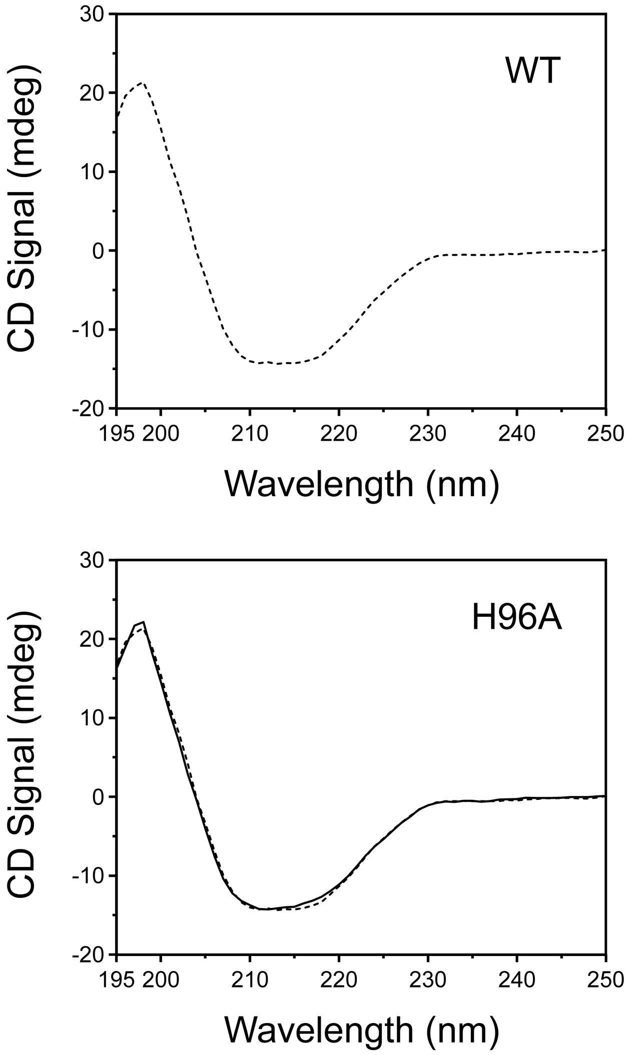

Supplementary Figure 4. Secondary structure assessment of TIA-1 His-to-Ala mutants. Far-UV CD spectra of TIA-1 RRM2,3 constructs at a concentration of 10 µM. The profiles of H94A, H96A and H94A H96A mutants are presented as a solid line superimposed on the spectrum of the WT protein (dashed line) for comparison purposes.

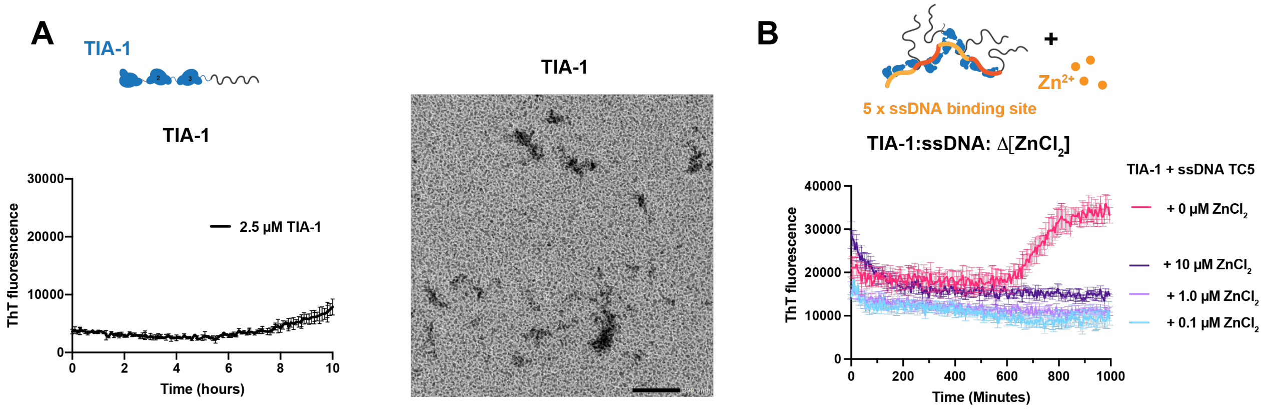

Supplementary Figure 5. Control experiments demonstrating (A) no significant TIA-1 aggregate formation in the absence of added co-factors and (B) ability of substoichiometric amounts of Zn^{2+} to suppress TIA-1 aggregate formation. Shown are Thioflavin T fluorescence assays monitoring the formation of ThT positive aggregates and TEM image of the final sample. All assays include 2.5 μM TIA-1 either alone or in the presence of ssDNA TC5 and 0 to 10 μM $ZnCl_2$. Conditions: 20 mM HEPES pH 7.2, 50 mM NaCl, 15 mM Arginine 30 °C with shaking. 3 replicates. Errors represent S.D. Scale bare represents 200 nm.

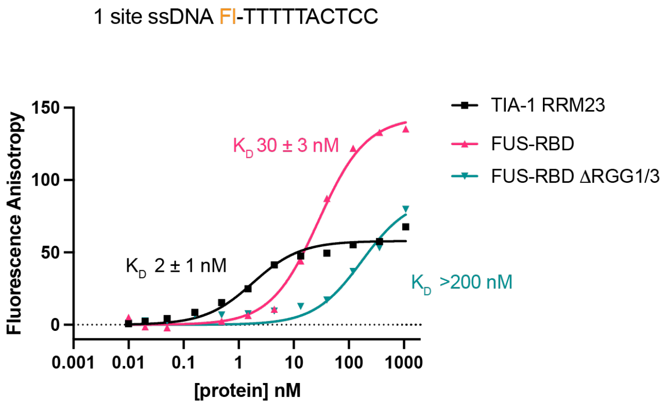

Supplementary Figure 6. FUS-RBD binding to TC DNA has a much lower affinity than that of TIA-1 RRM2,3. Fluorescence anisotropy (FA) of TIA-1 RRM2,3 and FUS-RBD constructs binding to ssDNA with one TIA-1 binding site (TC1) labelled with 5′-fluorescein (Fl-TTTTTACTCC) in 10 mM HEPES pH 7.2, 50 mM NaCl, 12 mM arginine, 1 mM (Shown is a single replicate. K_D is derived from two independent measurements).
